# 5G-enabled, battery-less smart skins for self-monitoring megastructures and digital twin applications

**DOI:** 10.1038/s41598-024-58257-7

**Published:** 2024-05-01

**Authors:** Charles Lynch, Ajibayo Adeyeye, El Mehdi Abbara, Ashraf Umar, Mohammed Alhendi, Chris Minnella, Joseph Iannotti, Nancy Stoffel, Mark Poliks, Manos M. Tentzeris

**Affiliations:** 1 Georgia Institute of Technology, School of ECE, Atlanta, GA 30308 USA; 2 Binghamton University, Binghamton, NY 13902 USA; 3 Sikorsky, a Lockheed Martin Company, Rochester, NY 14623 USA; 4 GE Global Research, Niskayuna, NY 12309 USA

**Keywords:** Engineering, Electrical and electronic engineering

## Abstract

With the current development of the 5G infrastructure, there presents a unique opportunity for the deployment of battery-less mmWave reflect-array-based sensors. These fully-passive devices benefit from having a larger detectability than alternative battery-less solutions to create self-monitoring megastructures. The presented ‘smart’ skin sensor uses a Van-Atta array design enabling ubiquitous local strain monitoring for the structural health monitoring of composite materials featuring wide interrogation angles. Proof-of-concept prototypes of these ‘smart’ skin millimeter-wave identification tags, that can be mounted on or embedded within common materials used in wind turbine blades, present a highly-detectable radar cross-section of − 33.75 dBsm and − 35.00 dBsm for mounted and embedded sensors respectively. Both sensors display a minimum resolution of 202 $\upmu$-strain even at 40^∘^ off-axis enabling interrogation of the fully-passive sensor at oblique angles of incidence. When interrogated from a proof-of-concept reader, the fully-passive, sticker-like mmID enables local strain monitoring of both carbon fiber and glass fiber composite materials. The sensors display a repeatable and recoverable response over 0–3000 $\upmu$-strain and a sensitivity of 7.55 kHz/$\upmu$-strain and 7.92 kHz/$\upmu$-strain for mounted and embedded sensors, respectively. Thus, the presented 5G-enabled battery-less sensor presents massive potential for the development of ubiquitous Digital Twinning of composite materials in future smart cities architectures.

## Introduction

With the recent advances in the Internet of Things (IoT) Technologies and the proliferation of 5G wireless systems, there is an expected total of 22 billion IoT devices to interact with these wireless networks by 2025^1^. These devices can enable next-generational wireless cyberphysical systems (CPSs) which operate by dispersing sensor nodes throughout a physical environment that is desired to be monitored. These CPSs enable real-time, data-driven modeling of physical systems for optimized operation to reduce costs. However, a proportional number of batteries would be required to power these sensors, thereby, dramatically increasing manufacturing, environmental, and maintenance costs of CPSs, impeding the widespread adoption of these systems. Backscatter-based wireless communication through the use of radio frequency identification (RFID) and millimeter wave identification (mmID) provides a wireless solution that is highly scalable, due to the ultra-low-cost, and fully-passive operation, removing the need for costly batteries. RFIDs and mmIDs both utilize the concept of backscattering an impinging interrogating signal and encoding sensor information on this reflected signal back to a gateway for further processing. These backscattering modules have been utilized for a wide variety of sensing applications including relative humidity, temperature monitoring, and local strain sensing^2–4^.

While a vast amount of passive RFID research has been conducted with ultra-high frequency spectral bands, these wireless systems suffer due to the lower available equivalent isotropic radiated power (EIRP) as well as a crowded spectrum with multiple interferers hindering operation of fully-passive, chipless wireless CPS. Thus, by shifting to a mmID-based CPS, one benefits from the wide bandwidth of operation, and increased EIRP to enable dense spatial multiplexing of these mmIDs throughout an environment. A chipless, fully-passive, additively manufactured ‘smart skin’ relative humidity sensor displayed reading ranges up to 30 m by taking advantage of the high EIRP in the 5G/mmWave bands^5^. Potential use cases of these wireless sensors is in the construction of Digital Twins for Structural Health Monitoring (SHM) of structures such as wind turbine blades, civil structures, or aircraft. The data captured in these twins can be used to optimize maintenance and overhaul of structures as well as to reduce the likelihood of catastrophic accidents by providing prior warning when operational loads exceed design stresses. In this application space, there have been both wired and wireless approaches for these SHM Digital Twinning systems. The wired approaches that have been reported in literature include the use of arrays of resistive strain gauges throughout a composite material^6,7^, carbon nanotube-based multi-axis strain sensing^8^, piezoelectric sensors that convert the mechanical deformation brought on by strain into a measurable voltage waveform^9,10^, and a Fiber–Bragg grating designed to monitor local strain and temperature^11^. While these sensing techniques provide reliable readings, these wired sensing systems leave much to be desired from an implementation stand-point. With difficulties in the electrical routing of the sensors throughout the composite structure, especially under scenarios where the structure is intended to move such as in the case of a helicopter blade or wind-turbine blade, and the powering of data acquisition circuits translate the sensor response into an electrical signal to be the input of the Digital Twin model, these wired sensing systems lack the practicality that a wireless sensing system brings. Fully-passive wireless SHM promises a completely energy autonomous and easily implementable solution to form these next-generation Digital Twinning systems. Through the use of RFID-based sensors which are designed either by recording resistive strain gauge information in the data of the backscattered signal^12^ or using the antenna element to tune the amplitude and frequency based on a mechancial deformation^13–15^. However, these RFIDs all utilize a chip-based transponder that has the two limitations, namely the required power received at the tag in order for the RFID chip harvest the impinging energy and backscatter the sensor information back, thereby reducing operational range, and the difficulties of embedding such sensors into a composite material during the fabrication process due to the thermal curing needed to form glass-fiber composite materials. Therefore, to overcome these limitations, completely chipless designs are prefered. Chipless-based RFIDs that have been presented in the literature include the use of loading a flexible textile-based patch antenna, a loading structure on a patch antenna for 2-D strain sensing, loading of a metamaterial-based resonator, and an embeddable resonating cavity-based strain sensors^4,16–18^. All of these works operate through RF resonating structures which when experiencing local geometric deformation corresponds to a change in resonating frequency of the structure, due to the proportionality of the resonant frequency and the physical dimension of an RF structure. However, the reading ranges of these sensors fall on the order of a few centimeters.

**Figure 1:**
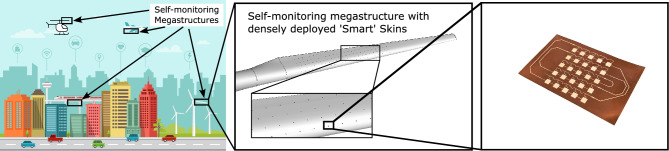
Diagram of the ‘smart’ city infrastructure with dense deployment of the proposed ‘smart’ skin strain sensor for SHM with a magnification of proof-of-concept application on wind turbine blades.

In order to enable a stand-off-range wireless CPS for structural health monitoring with the ubiquitous operation, the authors propose a Van-Atta ‘smart skin’ reflectarray-based strain sensor. The concept behind this highly scalable, ultra-low-cost, and fully passive wireless strain sensor is displayed in Fig. 1 with proposed self-monitoring megastructures highlighted and densely deployed.

This compact fully-passive flexible ‘smart skin’ strain sensor form factor enables both mounted and embedded deployment of the sensor. The proposed 5G/mmWave strain sensor prototypes for wind blade turbines displayed a sensitivity of 7.55 kHz/$\upmu$-strain and 7.92 kHz/$\upmu$-strain, for mounted and embedded deployed sensors at 1 m away from the proof-of-concept interrogator, respectively. Thus, the work presented displays the advantage of reflect-arrays for local strain monitoring and utilizing the available 75 dBm of EIRP at 5G/mmWave stand-off ranges of 35 m are envisioned.

## Experiments, results, and discussion

### Fully-passive wireless interrogation of smart skins

When considering fully-passive wireless interrogation, a primary challenge arises from extracting the sensor response in the signal observed at the reader. This signal is comprised of the environmental scattering, the structural scattering of the sensor, the transmitting signal from the interrogator, all acting as interference that can mask the sensor response, and finally, the desired encoded scattering of the sensor mapping to the local strain experienced by the sensor. To mitigate these sources of interference, the interrogator and sensor can take advantage of a cross-polarized interrogation, having the transmitting and receiving polarization be orthogonal to each other, thereby reducing the power level of all the sources of noise while maintaining the same power level of the sensor response. Along with system-level choice, selecting 5G/mmWave frequency bands for interrogation presents several advantages. Firstly, this allows for highly directive interrogating antennas and higher EIRPs with respect to UHF RFID systems, enabling long-range interrogation by increasing the available power to be transmitted in the link budget. The received power in the link budget of this system is expressed by

1 $$\begin{aligned} P_{r} = P_{i} {A_{i}}^{2} {e_{i}}^{2} D_{t}(\theta ) \frac{\sigma _{t}}{4\pi {\lambda }^{2} {R}^{4}} \end{aligned}$$

where $P_{i}$, $A_{i}$, $e_{i}$, $D_{t}(\theta )$, $\lambda$, and *R* are the transmitted power of the interrogator, physical aperture area of the transmitting and receiving antennas of the interrogator (assumed to be symmetrical), aperture efficiencies of the transmitting and receiving antennas of the interrogator, the radiation pattern of the tag with respect to the angle of incidence, the wavelength of electromagnetic radiation, and the range of the mmID from the interrogator. The $\sigma _{t}$ or the radar cross section (RCS) of the tag, which is proportional to the power level received by the sensor response, is described by

2 $$\begin{aligned} \sigma _{t} = \frac{4\pi A_{t} e_{t}}{{\lambda ^{2}}} \end{aligned}$$

where $A_{t}$ and $e_{t}$ are the physical aperture area of the mmID and the aperture efficiency of the mmID. Therefore, assuming the same physical area and efficiency and using the relationship between $\lambda$ and *f*, namely $f = c/\lambda$, the RCS of the tag—thus the detectability—would increase with $f^{2}$ displaying massive benefit of utilizing mmWave for chipless-based mmID applications in stand-off range scenarios. However, typical high-gain structures that would provide high detectability, suffer in angular coverage a challenge fully overcome by utilizing the proposed retro-directive Van-Atta reflectarray design. Thus, establishing the cross-polarized Van-Atta array as a prime candidate for ubiquitous strain sensing. Another practical challenge to overcome when interrogating these mounted and embedded wireless sensors for SHM of large composite structures is the scattering from the composite material itself. Two widely used composite materials are glass fiber and carbon fiber which have been manufactured for large turbines or helicopter blades. However, these two materials are vastly different electrically when considering wireless interrogation. Carbon fiber composites can be modeled as large electrical conductors due to their electrical conductivity and thus only allow for adhering the wireless sensors to the outer layer for wireless interrogation as most of the electromagnetic radiation is unable to penetrate through the composite material. Glass-fiber composites can be modeled as a lossy dielectric material. Thereby, allowing for embedded sensors that can be wirelessly interrogated as electromagnetic radiation can travel through the material. Due to the flexibility and small form factor that can be achieved at 5G/mmWave, the proposed strain sensor can either be embedded or mounted which makes it a great candidate for achieving fully-passive wireless interrogation. Furthermore, to overcome the large structural mode of these composites, the retro-directive behavior of the reflectarray enables to off-axis interrogation in order to decouple this diffractive reflection from the incident wave away from the receiving antenna as this initial structural reflection will be directed away from the receiver dictated by Snell’s law. Thus, establishing 5G/mmWave retro-directive-based sensors as a paradigm-changing solution for fully-passive wireless SHM due to the increased reading ranges possible and the ability to overcome the main sources of noise in fully-passive wireless sensing.

### Flexible, chipless, mmWave strain smart skin

**Figure 2:**
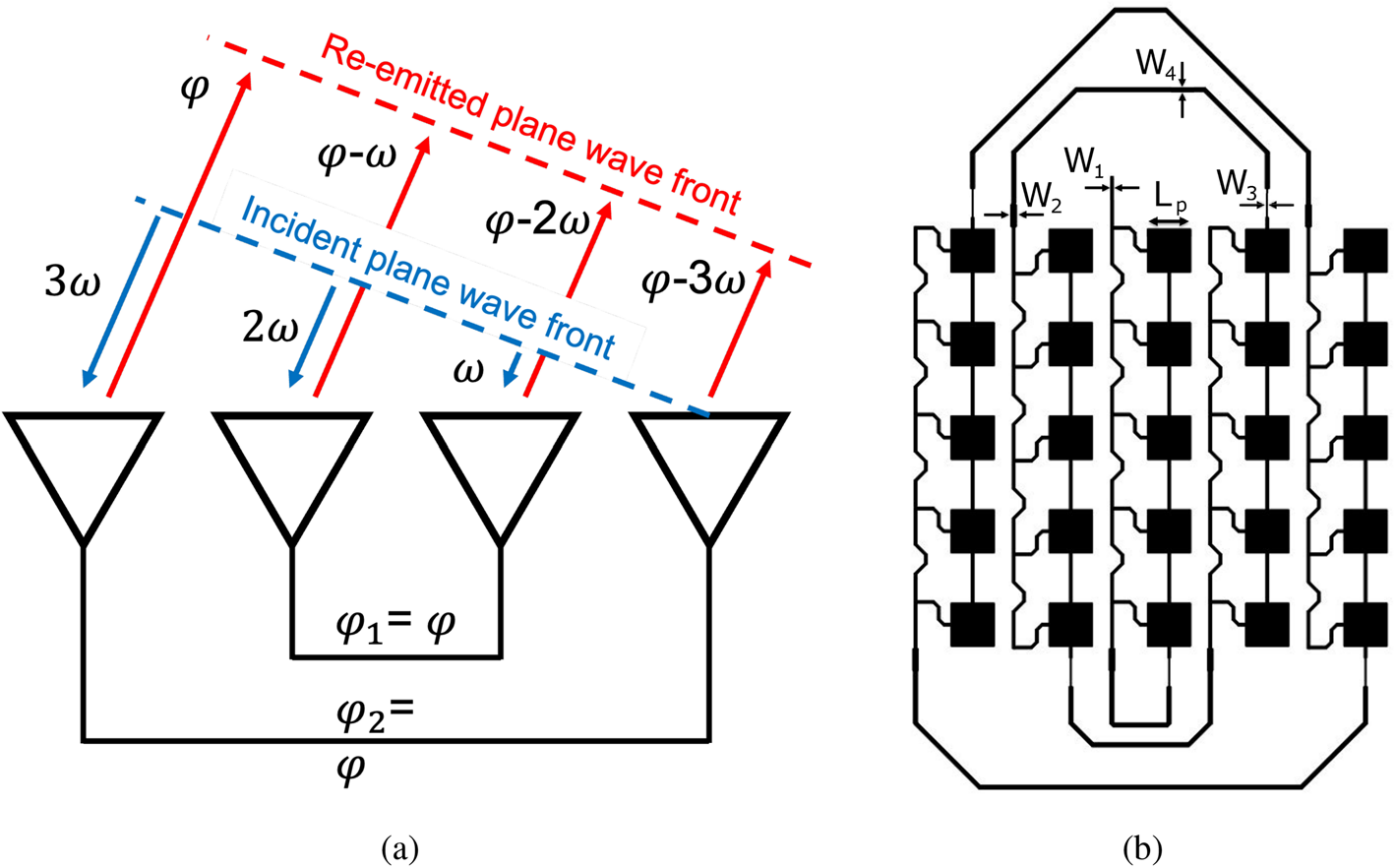
(**a**) Diagram of the retro-directive operation of the Van-Atta reflectarray. (**b**) Schematic of the fully-passive 5G/mmWave strain sensor design with $L_{p}$ = 3.41 mm, $W_{1}$ = 0.2 mm, $W_{2}$ = 0.422 mm, $W_{3}$ = 0.062 mm, and $W_{4}=$ 0.303 mm and total foot print of 70 mm $\times$ 45 mm.

As aforementioned, the fully-passive, mmWave ‘smart’ skin sensor utilizes a cross-polarized Van-Atta array-based-mmID. This mmID is comprised of five cross-polarized $5 \times 1$ patch antenna arrays and spaced $\frac{\lambda }{2}$ at the operational frequency of 24.125 GHz and designed on 0.127 mm thick, low-loss flexible substrate Kapton HN ($\epsilon _{r} = 3.14, \tan (\delta )=0.0014$). By placing the arrays with this spacing, the maximum angular coverage is $\pm \, 90^{\circ }$, but due to the patch antenna element having a maximum beam width of $\pm \, 60^{\circ }$ about boresight, this is the maximum ideal off-axis interrogation angle that the Van-Atta can support. As previously mentioned, this off-axis interrogation is crucial to reduce the amount of environmental and structural mode reflection received at the receiver antenna during wireless interrogation of fully passive ‘smart’ skin sensors. To minimize the footprint of the sensor of the Van-Atta sensor, the single arrays were flipped by 180^∘^ and the transmission line electrical length was correspondingly 180^∘^ out of phase to properly re-radiate in phase to the direction of arrival enabling stand-off range interrogation. The motivation of minimizing the footprint of the Van-Atta is to mitigate any mechanical weaknesses the mounted and embedded implementations might cause while the composite material is under stress. Additionally, as presented in^5^, the $5\times 5$ design enabled long reading ranges up to 30 m for humidity sensing and therefore, was selected as the optimal size for the wireless strain sensor. The concept behind this retro-directive behavior can be viewed in Fig. 2a as well as the schematic of diagram of the fully-passive, sticker-like Van-Atta array strain sensor in Fig. 2b.

**Figure 3:**
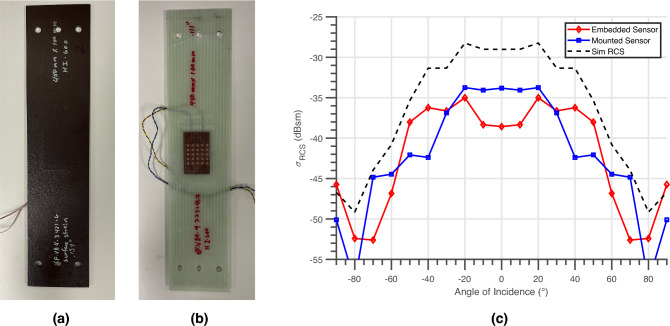
(**a**) The mounted 5G/mmWave ‘smart’ skin sensor deployed on carbon fiber composite material for local SHM. (**b**) The embedded 5G/mmWave ‘smart’ skin sensor deployed on glass fiber composite material for local SHM. (**c**) Simulated and measured radar cross-section versus angle of incidence of the interrogation of the standalone simulation model, the mounted, and embedded ‘smart’ skin wireless strain sensor.

The RCS of both mounted and embedded Van-Atta array, displayed in Fig. 3a,b respectively, over a ± 90^∘^ interrogation angle was measured utilizing a vector network analyzer (VNA), two identical 20 dBi horn antennas oriented in a cross-polarized configuration 1 m away from the interrogating horns. A 12^″^ in.-diameter metal sphere RCS standard was measured to calibrate and confirm the RCS of the proposed fully-passive Van-Atta array strain sensor. The simulated and measured RCS versus angle is displayed in Fig. 3c and the measured mounted and embedded wireless strain sensor displays good agreement to that of the standalone simulated RCS Van Atta array. With less than a 5 dB difference of the peak RCS between the simulated RCS and the measured RCS of both sensors fabricated, this establishes the ability to deploy these sensors throughout the composite materials with a manageable impact on the detectability of the sensor. Additionally, the measurement confirms the retro-directive response of the proposed sensor and enables fully-passive wireless interrogation even with highly oblique angles of incidence to the strain sensor with a peak RCS of − 33.75 dBsm and − 35.00 dBsm of the mounted and the embedded sensor respectively at ± 20^∘^.

**Figure 4:**
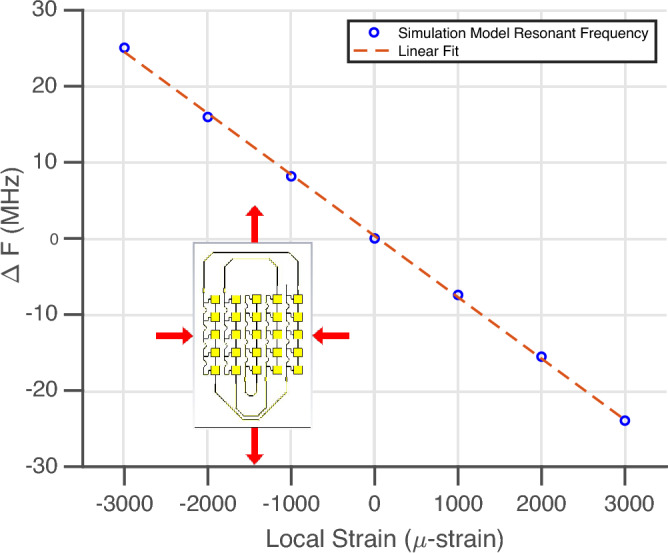
Uniaxial strain simulations of 5G/mmWave ‘smart’ skin strain sensor.

In order to determine the expected sensitivity of the fully-passive strain ‘smart’ skin, the simulation model was scaled based on uniaxial strain in the vertical dimension, and using the Poisson coefficient of Kapton HN, the off-axis scaling was applied to both x- and z-axis and the resonant frequency of the Van-Atta array was recorded from ± 3000 $\upmu$-strain were conducted and displayed in Fig. 4 in steps of 1000 $\upmu$-strain with the simulation model inset and arrows denoting the orientation of the scaled-based strain applied to the model.

As expected the relationship between local deformation, and thus the local strain experienced by the sensor, and frequency have a negative slope due to the inverse relationship between wavelength and frequency of operation in wireless design. A linear fit of this change in resonant frequency was made and the chipless, 5G/mmWave strain sensor exhibits an estimated sensitivity of 8.05 kHz/$\upmu$-strain. Thus, the proposed strain sensor will provide a fully-passive mechanism of providing local strain information for the structural health monitoring of the composite materials.

**Figure 5:**
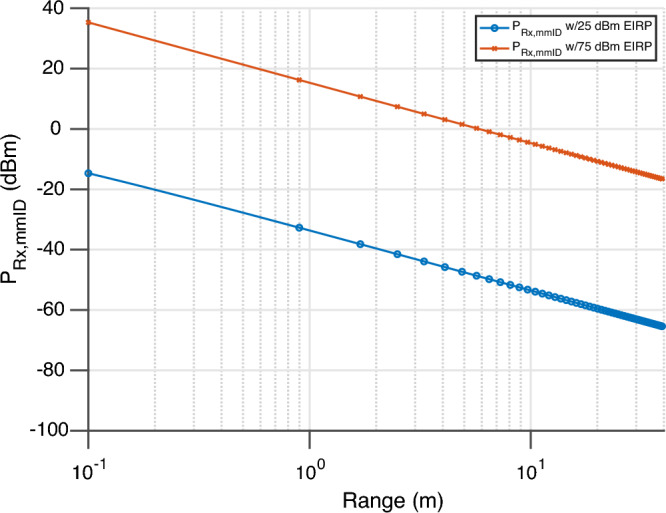
Received power impinging onto the fully-passive mmID with the current PoC reader and a future reader with an EIRP of 75 dBm.

Having characterized the static RCS of each realization of the ‘smart’ skin sensor, a link budget analysis will be presented to determine maximum operational range of the proposed fully-passive wireless strain sensor. For the link budget of this system, four scenarios were considered including: the same designs with the current PoC reader specifications for the relfect array interrogated at 20^∘^ and at 40^∘^ angle of incidence and a future system with the same sensitivity level with an EIRP of 75 dBm—the maximum allotted EIRP in 5G/mmWave frequencies—with the same angles of incidence to the fully-passive strain sensors. The first link that was analyzed was the link from the reader to the wireless strain sensing mmID. The power impinging onto the mmID is described by

3 $$\begin{aligned} P_{Rx,mmID} = P_{Tx}+G_{Tx}+10 n_{f_{o}} \log _{10}\left( \frac{\lambda }{4 \pi R}\right) \end{aligned}$$

where $P_{Tx}$, $G_{Tx}$, $n_{f_{o}}$, $\lambda$, and *R*, are the transmitting power in dBm, the gain of the transmitting antenna in dBi, the channel path loss exponent which is dependent on the channel conditions at the transmitting frequency of the reader and is unit-less, the wavelength at the transmitting frequency of the reader in meters, and the distance from the reader to the mmID in meters. A plot of this for all link budget scenarios is displayed in Fig. 5.

**Figure 6:**
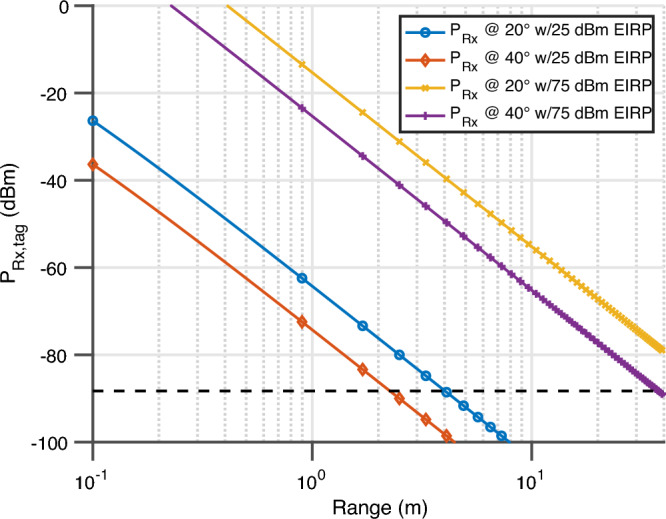
Received power of the sensor response of the PoC reader with 25 dBm EIRP and a future reader with 75 dBm EIRP with the sensitivity level labeled.

After impinging on the mmID, the structural mode will scatter away and the retrodirective sensor response is scattered back towards the reader in the orthogonal polarization. The power of this sensor response received by the reader is described by

4 $$\begin{aligned} P_{Rx,Reader} = P_{Rx,mmID} +\sigma _{RCS}\left( \Theta ,\Phi \right) +10 n_{f_{o}} \log _{10}\left( \frac{\lambda }{4 \pi R}\right) +G_{LNA} - L \end{aligned}$$

where $\sigma _{RCS}$, $\Theta$, $\Phi$, $G_{LNA}$, and *L* are the radar cross-section of the mmID in dBsm which is a function of the angle of incidence, the horizontal angle of incidence in ^∘^, the elevation angle of incidence in ^∘^, the gain of the LNA in dB, and the losses due to cables in dB. The final piece to the link budget analysis is the receiver sensitivity which is described by

5 $$\begin{aligned} S_{Rx} = -173.8 + 10\log _{10}\left( F_{samp}\right) +NF+SNR_{min}+G_{LNA} \end{aligned}$$

where the constant term, $F_{samp}$, *NF*, $SNR_{min}$ are the thermal noise spectral density in dBm per Hz, the sampling frequency of receiver Hz, and the minimum signal to noise ratio needed for accurate sensing of resonant frequency—therefore local strain—in dB. This received power of the implemented sensor for each of the four scenarios is displayed in Fig. 6. In this scenario, $F_{Samp}$, *NF*, $SNR_{min}$, and $G_{LNA}$ are set to 1 kHz divided by the number of frequency points (5001 points) as this is one observation of the sensor, 4.5 dB, 43 dB, and 45 dB, respectively. The sample frequency and the noise figure were taken from the PoC reader setup and the high SNR term was chosen based on the worst case link budget of the PoC reader for an angle of incidence of 40^∘^ while desiring to monitor the local strain information of the composite materials. This minimum SNR term was chosen to provide the require frequency resolution and noise observed from the sensor characterization section with a worst case error of 202 $\upmu$-strain. If another system required less $\upmu$-strain resolution, the term can be relaxed and therefore increase the overall range of the sensor.

Based on the link budget analysis, the maximum reading range of the current PoC system is a mere 1.15 m for the worst case angle of incidence of 40^∘^. However, while maintaining the same sensitivity and utilizing the available EIRP of 5G/mmWave frequencies (75 dBm, a maximum reading range in excess of 35 m is envisioned. Thus the presented link budget presents a theoretical limit of the proposed fully-passive scalable local strain sensing system.

### Benchmarking of deployed fully-passive mmWave strain sensing

**Figure 7:**
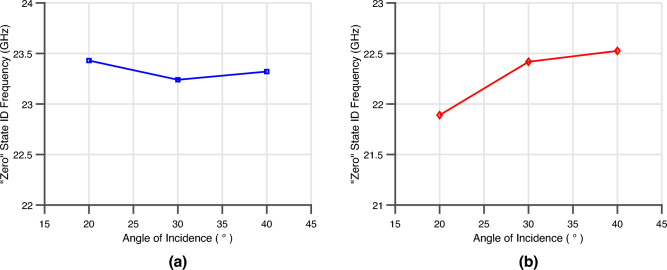
(**a**) The ‘zero’ state ID frequency of mounted 5G/mmWave ‘smart’ skin sensor at different angles of interrogation. (**b**) The ‘zero’ state ID frequency of embedded 5G/mmWave ‘smart’ skin sensor at different angles of interrogation.

The designed 5G/mmWave was then either mounted on the carbon fiber composite or embedded in the glass fiber composite samples for bench-marking of the wireless strain sensing ‘smart’ skin. Each of these composite materials is used for different applications. For this research, the target application for carbon fiber reinforced polymer material was composite rotorcraft structures. Similarly, the target application for the glass fiber composite material was wind turbine blades. With the use of the fully passive strain sensor, it will be possible to determine the localized strain state of the composite. This capability will be useful as a structural health monitor to monitor for repair or replacement and fitness for service. It could also be used as part of a closed loop feedback system for blade control during high winds. To characterize the both sensor types, two proof-of-concept samples—one carbon fiber and one glass fiber composite—were machined and assembled with dimensions $100\times 450 \times 2.8$ mm with the sensor centered in the middle. These specific dimensions were chosen due to the specific clamps and Instron utilized for the tensile loading of the samples. The mounted sensor also has a protective coating to seal out any humidity for practical use in a real-world system and the embedded sensor is placed in the middle layer of the glass fiber composite-approximately 1.4 mm in the composite. The composite was placed at a depth of 1.4 mm to keep the material structurally symmetric and allow for strain monitoring in the center of the glass fiber DUT. Furthermore, the embedded ‘smart’ skin had holes drilled in the substrate surrounding the Van Atta array patch with the intent to improve the strain transfer from the composite material to the flexible Van Atta array strain sensor. The sensor was then interrogated by a proof-of-concept reader comprised of a VNA, two identical linearly polarized 20 dBi horn antennas configured in a cross-polarized configuration, a transmitting power − 5 dBm feeding the transmitting horn antenna, and a PTFE dielectric lens to provide a total gain of 30 dBi—thereby constituting an EIRP of 25 dBm, a polarizer to utilize the same PTFE lens for the transmit and receive channels. This interrogator signal was then received by the fully-passive strain sensor and scattered back towards the reader with the frequency encoded local strain information. This signal was received through the same lens, passed through the polarizer, and received by the cross-polarized receiving horn antenna. This received signal was then amplified by 40 dB low noise amplifier (LNA) to increase the reader sensitivity and then sampled on port 2 of the VNA to record the sensor response. Prior to the mechanical loading measurement being conducted, the ‘zero’ state frequency of each sensor was obtained. This is to calibrate out the angle-induced frequency shift in the transfer function obtained and estimated sensitivity. The differences of the ‘zero’ state ID frequency of the mounted and the embedded 5G/mmWave ‘smart’ skin can be attributed to the differences in material stack up. With the mounted sensor having a environmental film laminated to cover and protect the sensor from the harsh environments caused a slight loading of the resonant frequency of the Van-Atta array. The embedded implementation has a larger static downshift in frequency due to the permittivity of the glass-fiber composite material. However, both Van-Atta arrays still maintained retro-directive responses at their ‘zero’ state frequencies as displayed in the previous section. The angular steps that were interrogated were from 20^∘^ to 40^∘^ in steps of 10^∘^ with the proof-of-concept interrogator. The ‘zero’ state frequency for each mounted and embedded sensor is displayed in Fig. 7a,b. Through interrogation with a narrow-band signal, the reader can to differentiate and identify the static frequency shift due to the angle of incidence of both sensor types.

**Figure 8:**
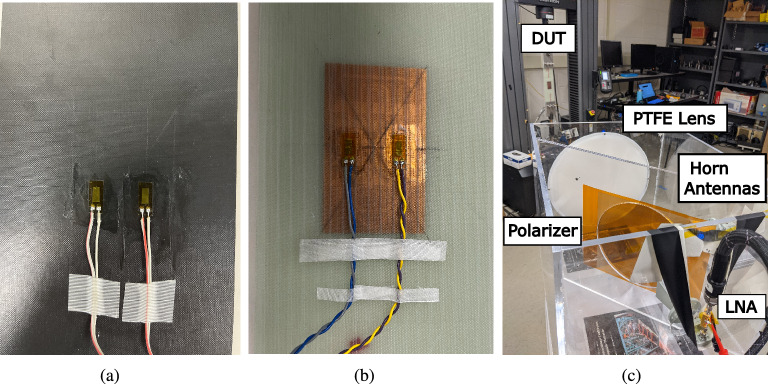
(**a**) Backside view of mounted ‘smart’ skin DUT with two glued wired strain gauges displayed. (**b**) Backside view of embedded ‘smart’ skin DUT with two glued wired strain gauges displayed. (**c**) Experimental setup of the cyclic loading of the embedded ‘smart’ skin sensor with the angle of incidence of 20^∘^ and at a distance of 1 m.

After characterizing the angle of incidence variation, the mounted and embedded sensors were measured under a loaded condition at each angle of incidence. To verify the sensitivity, recover-ability, and repeatability of each 5G/mmWave ‘smart’ skin each device under test (DUT) was subjected to 2 cycles of 3000 $\upmu$-strain that would reach maximum loading every 10 min with an idle time of 2 min at the start of the recording time. In Fig. 8a,b, the back view of each of the assembled DUTs is displayed and shows the industry-wired strain gauges that were utilized. The measurements were done with the DUT 1 m away from the proof-of-concept reader and an angle of incidence of 20^∘^–40^∘^ in steps of 10^∘^ to demonstrate the impact of highly oblique angles of incidence to sensor performance with this experimental setup shown in Fig. 8c.

**Figure 9:**
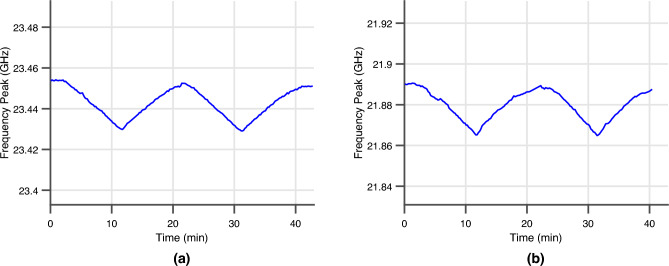
(**a**) The resonant frequency of mounted 5G/mmWave ‘Smart’ skin sensor under cyclic loading. (**b**) The resonant frequency of embedded 5G/mmWave ‘smart’ skin sensor under cyclic loading.

After time-gating the received response of the VNA sweep, the resonant frequency of the sensor for the mounted and the embedded sensor was tracked throughout the cyclic mechanical loading. The frequency shift of the mounted and embedded sensor at an angle of incidence of 20^∘^ throughout the cyclic loading measurement is displayed in Fig. 9a,b, respectively.

**Figure 10:**
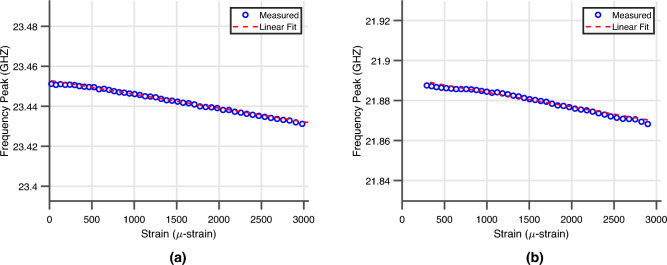
(**a**) The transfer function of the fully-passive 5G/mmWave ‘smart’ skin sensor mounted on the carbon fiber composite DUT. (**b**) The transfer function of the fully-passive 5G/mmWave ‘smart’ skin sensor embedded inside the glass fiber composite DUT.

From these measurements, both the mounted and embedded wireless ‘smart’ skin strain sensors displayed the expected inverse relationship between local strain and resonant frequency of the sensor as the simulation model. Using the ground truth wired foil strain sensors that were mounted to the back of each DUT, the transfer function of each sensor was created with a linear fit between the change in resonant frequency and the deformation experienced by each sensor. The transfer function of each sensor of the first loading cycle is displayed in Fig. 10a,b for the mounted and embedded sensor respectively.

**Figure 11:**
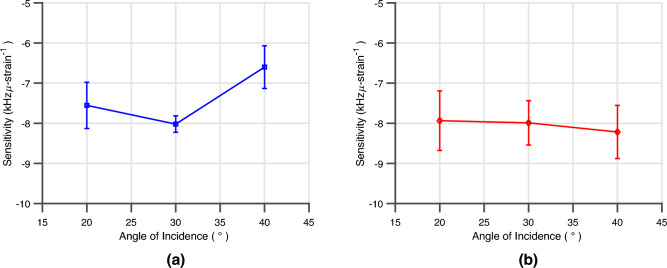
(**a**) The sensitivity of the fully passive 5G/mmWave ‘smart’ skin sensor under cyclic mechanical loading mounted on the carbon fiber composite DUT. (**b**) The sensitivity of the fully passive 5G/mmWave ‘smart’ skin sensor under cyclic mechanical loading embedded inside the glass-fiber composite DUT.

This experiment was then repeated for each angle of incidence previously and the resulting linear fits for each loading and de-loading cycle were calculated to estimate the sensitivity of the fully-passive sensors versus angle of incidence. The average sensitivity with standard deviation bars for the mounted and embedded fully-passive 5G/mmWave strain sensor for each of these measurements is displayed in Fig. 11a,b. The average sensitivity of the 7.55 kHz/$\upmu$-strain and 7.92 kHz/$\upmu$-strain closely match the sensitivity of the expected sensitivity of the simulation model. The maximum bounded average error at an interrogation angle of 40^∘^ of the sensors was calculated to be 202 $\upmu$-strain and 180 $\upmu$-strain for the mounted and embedded sensors, respectively. Thus, the sensor provides relatively high-precision local strain monitoring with a worst-case resolution of 202 $\upmu$-strain even at oblique angles of 40^∘^.

Both sensors display a highly repeatable and recoverable response due to the low deviation of each sensor throughout the cyclic loading. Table 1 displays the state of the art of various reported fully passive wireless strain sensors. Firstly, when compared to the other presented works, this work displays a superior sensitivity to all works expect for the cantilever design and a FSS metasurface sensor. However, due to the retro-directive behavior of the Van-Atta array-based sensor, the proposed work also enables highly oblique angles of interrogation—± 20^∘^–40^∘^—without degradation to sensor sensitivity, a massive improvement relative to that of the patch cantilever sensor and the FSS metasurface sensor with only one angle of interrogation characterized. Furthermore, when comparing against the FSS metasurface, this sensor utilizes a transmission based sensing, requiring bi-static and RF transparent material for sensing. Thus making the FSS metasurface-based sensor less practical and robust than the proposed reflect array-based ‘smart’ skin strain sensor. Furthermore, the metasurface requirement for bi-static interrogation increases the necessary wireless infrastructure for sensing, something the proposed Van-Atta array-based strain sensor simplifies with the retro-directive response. Additionally, while the current reading range of the sensor is 1 m, through the development of the reader and taking advantage of the 75 dBm allotted to mmWave by the FCC, reading ranges in excess of 35 m are envisioned for stand-off range SHM.

**Figure 12:**
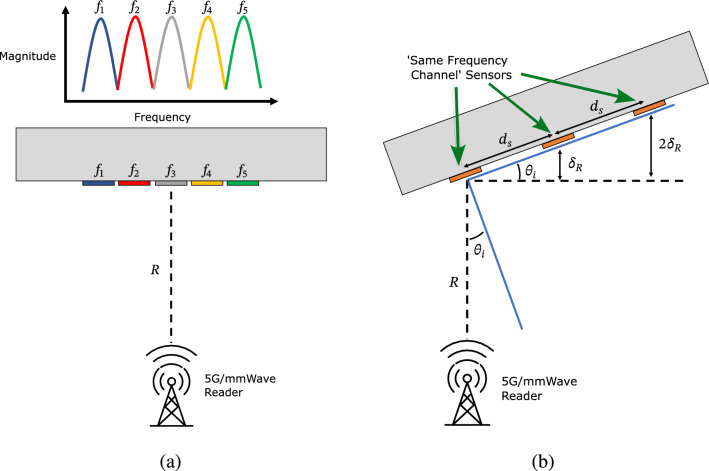
(**a**) Diagram of frequency multiplexed ‘Unit cell’ of fully-passive strain sensors. (**b**) Diagram of the spatial multiplexing of based on the angle of interrogation and spacing between ‘same frequency channel’ sensors.

For considering the dense deployment of these sensors to enable self-monitoring megastructures for SHM and Digital Twinning applications several considerations need to be made to determine the appropriate spacing of sensors. Due to the retro-directive behavior of the sensors only about the horizontal direction, the spacing estimations presented are for only the spacing in horizontal direction. However, the same presented analysis holds true if the proposed directive structures are rotated 90^∘^ for vertical spacing of the sensors. In general, the number of sensors interrogated simultaneously within the reader’s interrogating footprint is dependent on the bandwidth of the sensors based on a frequency multiplexing scheme. The proof-of-concept mmIDs display a half-power bandwidth of approximately 750 MHz, therefore, an 750 MHz channel is needed per sensor for a given range from the reader. Thus, within the typical 5G/mmWave available bandwidths of 4 GHz, one can interrogate 5 sensors simultaneously in the same range taking advantage of the different respective channels as shown in Fig. 12a. In addition to frequency multiplexing, spatial multiplexing can be employed to isolate sensors in the same frequency channel to efficiently reuse the frequency spectrum and further increase the number of sensors that can be simultaneously interrogated for a given bandwidth. Given the off-axis interrogation of the proposed reflectarray sensors, a spatial division based on the angle of incidence of sensors in the same plane can be easily achieved. The minimum spacing between two or more “same frequency channel” sensors to provide spatial isolation—the range resolution of a wireless interrogator—is given by the expression

6 $$\begin{aligned} \delta _{R} = \frac{c}{2BW}, \end{aligned}$$

where *c* and *BW*, are the speed of light and bandwidth of the reader. Furthermore, by leveraging the off-axis interrogation, the minimum horizontal spacing can then be calculated using the expression

7 $$\begin{aligned} d_{s} = \frac{\delta _{R}}{\sin (\theta _{i})} \end{aligned}$$

where $d_{s}$ and $\theta _{i}$ are the minimum horizontal spacing and angle of incidence with an image of the scenario displayed in Fig. 12b.

Therefore, assuming 4 GHz of bandwidth, the minimum spacing between sensors in range is 3.75 cm, and given an angle of incidence of 20^∘^, the minimum spacing horizontally to provide the radial separation of sensors operating in the same frequency channel is 10.81 cm. Therefore, assuming the sensors are multiplexed in frequency and are 4.5 cm in width, the same as the proposed sensor design, sensors can be spaced adjacent to each other for maximum spatial granularity of local strain monitoring in a horizontal row. Defining a “Unit cell” of sensors as a group of 5 frequency divided senors, one unit cell covers 7 cm $\times$ 22.5 cm area, greater than the minimum spacing needed between two sensors on the same channel. Therefore, resulting in a sensor density of 31.5 cm^2^ per “Unit cell”. Using the proof-of-concept reader antenna gain of 30 dBi—constituting a half-power beamwidth of 5^∘^—the diameter of the spotlight of illumination at 10 m, well within the maximum range of the wireless strain sensor can support, over a proposed self-monitoring megastructure is 0.93 m. Therefore, the maximum number of “Unit cells” that can be read over the diameter is 4, resulting in 20 individual sensors. This proposed spatial/frequency multiplexing scheme enables a suitable infrastructure for dense deployment of these fully-passive sensors for the realization of self-monitoring megastructures for a wide variety of digital twinning applications including SHM of bridges, buildings, and wind turbine blades.

**Table 1:** Comparison of reported state of the art in wireless passive strain sensors with * and ** denoting mounted and embedded wireless strain sensor respectively.

References	Structure	Freq.	Interrogation angle	Embedded	Sensitivity
^16^	Textile-based patch antenna	2.45 GHz	0^∘^	N/A	3.00 $\frac{\text {kHz}}{ \mu -strain}$
^17^	SRR	11.2 GHz	0^∘^	N/A	85 $\frac{\text {kHz}}{ \mu -strain}$
^19^	FSS metasurface	12.3 GHz	0^∘^	N/A	5.15 $\frac{\text {kHz}}{ \mu -strain}$
^20^	PIFA	915 MHz	0^∘^	N/A	0.55 $\frac{\text {kHz}}{ \mu -strain}$
^21^	RFID	915 MHz	0^∘^	N/A	0.73 $\frac{\text {kHz}}{ \mu -strain}$
^22^	Patch with cantilever	3.6 GHz	0^∘^	N/A	8.33 $\frac{\text {kHz}}{ \mu -strain}$
This work	Van-Atta Array	23.44*/21.89** GHz	± 20^∘^–40^∘^	Yes	7.55*/7.92** $\frac{\text {kHz}}{ \mu -strain}$

## Conclusion

In summary, the proposed 5G/mmWave-enabled system displays a wireless interrogation framework for the next-generation cyber physical system of large composite material SHM for self-monitoring megastructures and Digital Twin applications. The ‘smart’ skin reflectarray-based mmID proof-of-concept prototypes that were attached on/embedded in commonly used materials for wind turbine baldes displayed a highly detectable peak RCS of − 33.75 dBsm and − 35 dBsm for the mounted and embedded sensor respectively. Each sensor displayed a measured 10 dB beamwidth of ± 40^∘^ supporting highly oblique interrogation angles, overcoming the issue of the structural interference observed when interrogating at boresight. The ‘smart’ skin reflectarray-based mmID sensor requires no external power and each sensor was interrogated off-axis with a proof-of-concept reader under cyclic mechanical loading in order to evaluate the performance of the sensor. Both sensors displayed a repeatable, recoverable response with a sensitivity of 7.55 kHz/$\upmu$-strain and 7.92 kHz/$\upmu$-strain for mounted and embedded strain sensors respectively. The experimental results display on-par sensitivity to practical passive reflective-based strain sensor while being 1 m away from the proof-of-concept reader. Through further development of the reader, and by taking advantage of the 75 dBm EIRP in the mmWave spectrum, reading ranges in excess of 35 m are envisioned taking advantage of 5G/mmWave infrastructure. Thus the proposed system enables energy-autonomous, ubiquitous self-monitoring megastructures enabling optimal life-cycle management of wind turbine blades, smart buildings, and ‘smart’ bridge.

## Data Availability

The datasets used and/or analyzed during the current study available from the corresponding author on reasonable request. No human participants were involved in this study.
